# Prevalence and grade of diabetic peripheral neuropathy among known diabetic patients in rural Uganda

**DOI:** 10.3389/fcdhc.2022.1001872

**Published:** 2023-01-11

**Authors:** Dalton Kambale Munyambalu, Idania Hildago, Yves Tibamwenda Bafwa, Charles Abonga Lagoro, Franck Katembo Sikakulya, Bienfait Mumbere Vahwere, Ephraim Dafiewhare, Lazaro Martinez, Fardous Abeya Charles

**Affiliations:** ^1^ Department of Internal Medicine, Kampala International University-Teaching Hospital, Bushenyi, Uganda; ^2^ Department of Clinical Chemistry Laboratory, Kampala International University-Teaching Hospital, Bushenyi, Uganda; ^3^ Department of General Surgery, Kampala International University-Teaching Hospital, Bushenyi, Uganda; ^4^ Faculty of Medicine, Université Catholique du Graben, Butembo, Democratic Republic of Congo; ^5^ Department of Internal Medicine, Mbarara University of Science and Technology, Mbarara, Uganda

**Keywords:** diabetic peripheral neuropathy, prevalence and grade, Kampala, Uganda, diabetes mellitus

## Abstract

**Background:**

Diabetic peripheral neuropathy (DPN) is the most common complication of diabetes mellitus (DM). Approximately 50% of diabetic patients are estimated to develop DPN, depending on disease duration and diabetic control. Early diagnosis of DPN will avoid complications, including non-traumatic lower limb amputation, which is considered the most debilitating complication, as well as significant psychological, social, and economical problems. There is a paucity of literature on DPN from rural Uganda. This study aimed to deliver the prevalence and grade of DPN among DM patients in rural Uganda.

**Methods:**

A cross-sectional study that recruited 319 known DM patients was conducted in an outpatient clinic and a diabetic clinic at Kampala International University-Teaching Hospital (KIU-TH), Bushenyi, Uganda, between December 2019 and March 2020. Questionnaires were used to obtain clinical and sociodemographic data, a neurological examination was carried out to assess the DPN, and a blood sample was collected from each participant (for random/fasting blood glucose and glycosylated hemoglobin analyses). Data were analyzed using Stata version 15.0.

**Results:**

The sample size was 319 participants. The mean age of study participants was 59.4 ± 14.6 years and there were 197 (61.8%) females. The prevalence of DPN was 65.8% (210/319) (95% CI 60.4% to 70.9%), and 44.8% of participants had mild DPN, 42.4% had moderate DPN, and 12.8% had severe DPN.

**Conclusion:**

The prevalence of DPN at KIU-TH was higher among DM patients and its stage might have a negative impact on the progression of Diabetes Mellitus. Therefore, clinicians should consider neurological examination as a routine during assessment of all DM patients especially in rural areas where resources and facilities are often limited so that complications related to Diabetic mellitus will be prevented.

## Introduction

Diabetes mellitus (DM) globally spread and affects people of all ages and races (1). The International Diabetes Federation (IDF) reported, in 2021, that 537 million adults aged 20–79 years are currently living with DM, which represents 10.5% of the world’s population in this age group, with Africa being the part of the world where more than half the people with DM are undiagnosed (2). About 80% of diabetic patients live in low- and middle-income countries and almost 4 million people die of diabetes and its complications, with half of these people below the age of 60 years (1–3). The incidence of diabetic complications is expected to increase, and about 6.7 million adults are estimated to have died as a result of DM or its complications in 2021 (1, 2). DM affects a wide variety of neurological complications, which may involve the peripheral or autonomic nervous system, or both, mostly impairing the quality of life of patients, with impact on morbidity and mortality outcomes (4).

Diabetic peripheral neuropathy (DPN) corresponds to a type of nerve damage that typically affects the feet and legs, and sometimes affects the hands and arms in diabetic patients (5). DPN is the commonest diabetic neuropathy and must be diagnosed after the exclusion of other causes of polyneuropathy. Distal symmetric sensorimotor polyneuropathy is the most common type of DPN (5, 6). However, some patients might be asymptomatic. The condition seems to be irreversible and remains the most common chronic complication of DM (4–6). The real prevalence of DPN is not known and reports vary from 10% to 90% in diabetic patients, depending on the criteria and methods used to define neuropathy, and it has been reported that neurological complications occur equally in both type 1 diabetes mellitus (T1DM) and type 2 diabetes mellitus (T2DM) patients (6). The regional prevalence of DPN is 12.9% in North America and 3.2% generally in Africa (7). Approximately 50% of patients with diabetes are estimated to develop DPN, depending on disease duration and diabetic control (8).

Early diagnosis of DPN will avoid complications, the most debilitating of which is non-traumatic lower limb amputation, resulting in significant psychological and socioeconomic problems for the patient due to depression, societal stigmatization, and job loss due to the loss of a limb (6–8). In Africa there is widespread poverty and inadequate health insurance, which impedes the acquisition of efficient prostheses that would offset some of these problems. Even when overt gangrene has not yet complicated a foot ulcer, treatment of an ulcer drains the financial resources of the patient (9).

Few studies have been carried out in urban Uganda, but less is known in rural areas where patients are coming to seek help for their health in the advanced stages of the disease and with a high risk of complications related to DM (10). Therefore, the purpose of this study was to highlight the prevalence of DPN and its grade among DM patients in a rural southwestern Ugandan health facility.

## Methods

### Study design

This was a cross-sectional study, conducted from 13 December 2019 to 25 March 2020.

### Study site

The study was carried out at Kampala International University Teaching Hospital (KIU-TH) in the Department of Internal Medicine, located in Bushenyi District, in rural southwest Uganda.

### Study participants

Participants included all adult known DM patients (aged 18 years and above) who attended the medical outpatient patient department (MOPD), DM clinic, general outpatient department (GOPD), and private outpatient department (POPD) during the duration of the study, and who consented to participate. We excluded any patients with a mental disorder, patients who were unable to withstand an interview, patients who changed their consent, all pregnant women, newly diagnosed DM patients, and very sick DM patients admitted in the medical ward.

Participants were consecutively recruited. The sample size was of 319 patients using Leslie’s formula (1965), as shown in Equation 1:


$$N=\frac{Z^{2}p\;x\;q}{d^{2}}$$

**N** = desired sample size for population greater than 10,000.

**Z^2^** = standard normal deviation, assuming a 95% CI Z = 1.96.

**P** = proportion in the population estimated to have DPN in Uganda (Mulago Hospital, Kampala) = 29.4% according to Kisozi et al. (10).

### Data collection

Data were collected using a paper-based investigator-administered questionnaire that was designed in simple English and translated in local language for those who were unable to understand English.

Patients were given information about the study, and then written consent was sought and signed. Demographics (i.e., age, sex, address, marital status, education status), history of chronic illness, such as hypertension, kidney disease, and HIV infection status, and social habits, such as the use of alcohol and volume, smoking cigarette status and number of sticks per day, and adherence to medication were taken.

The body mass index (BMI) was calculated from a ratio of the patients’ weight in kilograms to the square value of the height in meters (kg/m^2^). Normal BMI was defined as< 24.9 kg/m^2^, overweight as 25 to 29.9 kg/m^2^, and obesity as ≥ 30 kg/m^2^ (WHO, 2000). Blood pressure was measured by using a manual sphygmomanometer, with appropriate cuff sizes for the patient arms being used. High blood pressure was defined as having a systolic blood pressure ≥ l40 mmHg or diastolic pressure ≥ 90 mmHg (European Society of Cardiology/European Society of Hypertension, 2018).

The physical/neurological examination was done. Pressure sensation was assessed using a 10-g monofilament (i.e., the Semmes–Weinstein monofilament test) at four of the ten standard sites of the sole of the feet (plantar base of the big toe, second and fifth toes, and at the heel), avoiding areas with callosity. Vibration sense was elicited using a 128-Hz turning fork at the big toe. Achilles deep tendon reflex was tested by using a standard patellar hammer.

Using a sterile disposable syringe and needle, 4 ml of blood was withdrawn from the anterior cubital fossa of each patient after cleaning the skin with a swab soaked in 70% alcohol. The blood sample was placed in a EDTA (ethylenediaminetetraacetic acid) purple container for random blood sugar (RBS)/fasting blood sugar (FBS) and glycosylated hemoglobin (HbA_1c_) analyses. Furthermore, RBS/FBS was screened using a Control D glucometer machine made in India (2018) by Haiden Technology with the manufacturer’s glucose sticks. The level of HbA_1c_ was screened using an Ichroma II Machine (2017) and the appropriated reagents for measuring HbA_1c_. Each study participant received a printed copy of their RBS/FBS and HbA_1c_ results.

The Neuropathy Disability Score (NDS) was used in assessing the grade of DPN for each patient. The NDS system is a tool of neuropathy evaluation score ranging from 0 to 10, which can also be used for assessment of severity of peripheral neuropathy by considering four parameters: vibration sense by using a 128-Hz tuning fork (0 = present, 1 = reduced/absent for each foot), temperature sensation by using a cold tuning fork (0 = present, 1 = reduced/absent for each foot), pin-prick sensation by a monofilament test (0 = present, 1 = reduced/absent for each foot), and ankle reflex/Achilles tendon reflex by using a patellar hammer (0 = normal, 1 = present with reinforcement, 2 = absent per side). Absence of neuropathy (normal) was considered when the score was from 0 up to 2. The grade of DPN disability was graded as follows: mild (scores: 3–5), moderate (scores: 6–8), and severe (scores: 9–10). The NDS is validated and is found to be 65% sensitive and 91% specific for diagnosing diabetic neuropathy (11). We used a 10-g monofilament test, patellar hammer, 128-Hz tuning fork/Hartman C 128 for the assessment of DPN, as described above.

### Data analysis

Data were captured in paper forms and entered into Epi Info™ 7.2, Microsoft Excel version 2010 and exported into Stata 15.0 for analysis. Data were processed accordingly and summarized using means for continuous variables or proportions for categorical variables.

For determining the prevalence of DPN at KIU-TH, we summarized data as frequencies and percentages, and 95% CIs were obtained for prevalence as an estimation measure.

### Ethics approval and consent to participate

The study was conducted after approval of Kampala International University-Research Ethics Committee (KIU-REC) under reference UG-REC-023/201939.

Written informed consent was obtained. Confidentiality for all the patients involved in the study was assured. People diagnosed with DPN were referred to the appropriate medical personnel.

## Results

Overall, 338 participants arrived at the MOPD, DM clinic, GOPD, and POPD at KIU-TH. Five participants were excluded from the study because three were newly diagnosed as having DM and two were very sick. A total of 333 participants met the inclusion criteria, and among them five declined to consent, and nine declined the examination and the blood sample collection. Finally 319 study participants consented, filled the study questionnaire, were examined, and blood samples taken during the study period and analyzed.

### Characteristics of the study participants

In **Table 1** below, majority (61.8%) of the participants were females, most (83.7%) of them were married and residing in a rural area (85.3%), with a mean age of 59.4 ± 14.6 years, and were agricultural workers by occupation (77.7%). In addition, most participants were T2DM (95.3%) on oral hypoglycemic agents (63.6%), overweight (55.8%) with a mean BMI of 26.26 ± 3.48 kg/m^2^, and poor glycemic control (53.9%), with a DM duration of less than 10 years (65.5%), i.e., with a mean duration of 7.33 ± 6.40 years. A few study participants were taking alcohol or had an Audit Score of 1 (10%), with a history of smoking (13.5%) and hypertension (50.2%).

**Table 1:** Baseline characteristics of the study participants.

Baseline characteristic	*N* = 319
Age (years), mean (± SD)	59.4 (± 14.6)
Female, *n* (%)	197 (61.8)
Rural residence, *n* (%)	272 (85.3)
Married, *n* (%)	267 (83.7)
Education level, *n* (%)	
Primary	129 (40.4)
Secondary	35 (10)
None	137 (42.9)
Occupation, *n* (%)	
Agricultural workers	248 (77.7)
Private business	22 (6.9)
Professional	22 (6.9)
Alcohol (Audit Score), *n* (%)	
Audit 1	35 (10)
Audit 2	31 (9.7)
Smoking, *n* (%)	43 (13.5)
DM duration, mean (± SD)	7.33 (± 6.40)
< 10 years	
≥ 10 years	209 (65.5)
Types of DM, *n* (%)	110 (34.5)
T1DM	
T2DM	15 (4.7)
Diabetic therapy, *n* (%)	304 (95.3)
Oral hypoglycemic agents	203 (63.6)
Insulin	41 (12.9)
Both oral hypoglycemic agents + insulin	57 (17.9)
Not on diabetic therapy	3 (5.6)
BMI (kg/m^2^), mean (± SD**)**	26.26 (± 3.48)
BMI categories (kg/m^2^), *n* (%)	
Normal (18.5–24.9)	103 (32.3)
Overweight (25.0–29.9)	178 (55.8)
Obese (≥ 30)	38 (11.9)
HbA_1c_ per cent, mean (± SD)	7.61 (± 2.47)
HbA_1c_ per cent, *n* (%)	
Good glycemic control (< 7.0)	147 (46.1)
Poor glycemic control (≥ 7.0)	172 (53.9)
Fasting glucose (mmol/l), mean (± SD)	10.35 (± 5.16)
Systolic blood pressure (mmHg), mean ( ± SD)	138.4 (± 19.77)
Diastolic blood pressure (mmHg), mean (± SD)	85.8 (± 12.95)
Hypertension, *n* (%)	160 (50.2)
HIV, *n* (%)	29 (9.1)

Audit: Alcohol Use Disorders Identification Test.

### Prevalence of DPN

**Figure 1:**
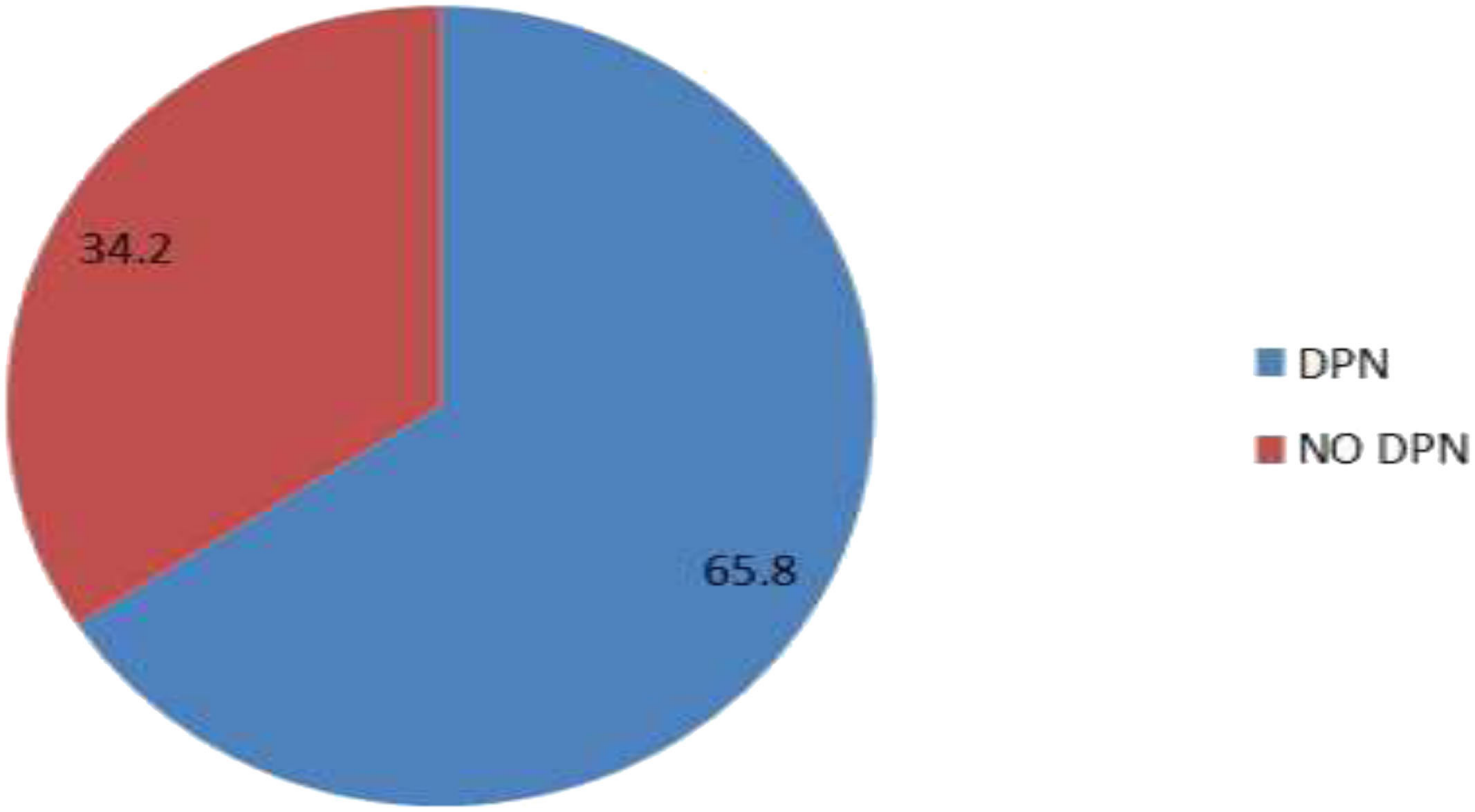
Prevalence of DPN among known DM patients attending KIU-TH.

The prevalence of DPN among adult diabetic patients attending Kampala International University-Teaching Hospital was 65.8% (210/319), (95% CI 60.4-70.9) by using the Neuropathy Disability Score (NDS) (**Figure 1**).

### Grade of DPN among known DM patients attending KIU-TH.

In our study, 210 (65.8%) participants had DPN, 44.8% had mild DPN, 42.4% had moderate DPN, and 12.8% had severe DPN.

## Discussion

### Characteristics of the study participants

The aim of this study was to determine the prevalence and grade of DPN among known DM patients in a rural setting of Uganda (KIU-TH, Ishaka in the Bushenyi District of southwestern Uganda). In this current study, most of the participants were female, agricultural workers, and from rural residency, with a mean age of 59.4 ± 14.6 years.

Garoushi et al. in a meta-analysis study conducted in the USA, the UK, France, Belgium, and South Africa (2018), and Morkid et al. in Bangladesh (2010), found that advanced age was significant with the occurrence of DPN (12). DPN develops progressively over months to years, and by the time the aging process is taking place there is a decrease in peripheral nerves function, mostly in lower extremities, with physical disabilities, gait disturbance, and falls.

### Prevalence of DPN

The overall prevalence of peripheral neuropathy among adult diabetic patients attending KIU-TH was 65.8% (95% CI 60.4% to 70.9%).

This study’s result is similar to the global prevalence of DPN (1). This could be explained by the fact that the global prevalence considers all the population by using a standard score (NDS) for assessing DPN. In a study carried out in India, the prevalence of DPN is lower than of this study (13). This disparity is due to the age of the participants, in which the previous study enrolled patients from age 30 years and above and they considered all the neurological complications in diabetic patients.

The prevalence of DPN in our study is similar to a study carried out in Morocco (14). The reason for the similarity could be because of the, almost, same social conditions as African countries and because methods used for the assessment of DPN were the same. This current study found a lower prevalence than a study carried out in Nigeria by Salawu et al. (15), because our study included all types of diabetes, whereas the other study considered only patients with T2DM.

The prevalence of DPN in this study was higher than those in studies carried out in Cameroon and Kampala (4, 10). In our study, the prevalence is higher probably because of the sample size, and the tools and criteria used for diagnosing DPN. In addition, the above study that was carried out in Kampala was conducted among newly diagnosed DM patients only.

### Grade of DPN

In our study, 210 (65.8%) participants had DPN, 44.8% (94/210) had mild DPN, 42.4% (89/210) had moderate DPN, and 12.8% (27/210) had severe DPN.

The study of Kazemi et al. in Iran (16) found that most of their participants had mild DPN and a few of them developed severe DPN, which corresponds to our findings as well, whereby the method used for the assessment of DPN were similar (NDS), with the same study design by considering a large number of DM patients. However, in Kampala, Kisozi et al. (10) got a larger number of patients with moderate DPN. The discrepancy could have been explained by the fact that in our study we used different tools for assessing DPN in the context of a rural setting and few of our participants had foot ulcers as a predictor of advanced DPN.

Vogt et al., in their study carried out in Tanzania (17), detected that severe DPN represented almost one-quarter of patients, which is different from our findings, because from their research, they compared only assessment tools for DPN without considering patient findings based on symptoms and signs. The NDS is an important tool for assessing DPN and constitutes a good predictor for risk of ulceration. Knowing DPN patients based on this score might help us to prevent patients from foot ulceration, diabetic foot, and other related complications that have a poor prognosis (18).

### Strengths and limitations

This is the first study concerning DPN in the southwest of Uganda and the sample size used was large to make a clear conclusion about our findings. Furthermore, the study did not use other diagnostic specific tests of DPN, such us corneal confocal microscopy and electrophysiological studies (e.g., nerve conduction studies, electromyography).

## Conclusion

The prevalence of DPN among known diabetic patients attending KIU-TH was higher, classified respectively in mild, moderate, and severe DPN, according to the number among DM patients. This might have a negative impact as DM is progressing. Therefore, for better prevention of other chronic complications, clinicians should consider neurological examination as routine during regular assessment of all DM patients, especially in rural areas where resources and facilities are often limited.

## Data availability statement

The raw data supporting the conclusions of this article will be made available by the authors, without undue reservation.

## Ethics statement

The studies involving human participants were reviewed and approved by Kampala International University-Research Ethics Committee. The patients/participants provided their written informed consent to participate in this study.

## Author contributions

DM conceived, designed the study, participated in the data collection, analysis, and drafted the manuscript. FA and YB analyzed the data and performed statistical tests. IH prepared and analyzed the collected blood samples. FS, BM, LM, ED, and CL assisted in the study conception/design and critically reviewed the manuscript. All authors approved the manuscript for publication.
